# Supplementary material for: Triple ionization and fragmentation of benzene trimers following ultrafast intermolecular Coulombic decay
Source: Nat Commun. 2022 Sep 10;13:5335. doi: 10.1038/s41467-022-33032-2 (PMC9464219; doi:10.1038/s41467-022-33032-2)
Supplement: Supplementary file 1 — Supplementary Information [file 41467_2022_33032_MOESM1_ESM.pdf]

Supplementary Information: Triple ionization and fragmentation of benzene trimers following ultrafast intermolecular Coulombic decay

Jiaqi Zhou,[1] Xitao Yu,[2] Sizuo Luo,[2] Xiaorui Xue,[1] Shaokui Jia,[1] Xinyu Zhang,[2] Yongtao Zhao,[1] Xintai Hao,[1] Lanhai He,[2] Chuncheng Wang,[2] Dajun Ding,[2,*] and Xueguang Ren[1,†]

[1]*MOE Key Laboratory for Nonequilibrium Synthesis and Modulation of Condensed Matter, School of Physics, Xi'an Jiaotong University, Xi'an 710049, China*

[2]*Institute of Atomic and Molecular Physics, Jilin University, Changchun 130012, China*

(Dated: August 24, 2022)

Contents

I. Supplementary Note 1 2

II. Supplementary Note 2 3

III. Supplementary Note 3 3

IV. Supplementary Note 4 6

* Electronic address: dajund@jlu.edu.cn

† Electronic address: renxueguang@xjtu.edu.cn

I. Supplementary Note 1

As shown in Supplementary Fig. 1, the Dalitz plots for two KER regions (6.0 - 6.8 eV and 6.8 - 9.0 eV) display different characteristics. The result for KER about 6.8 - 9.0 eV exhibits equilateral triangle pattern of the three-body dissociation channel with the highest density in the center of the Dalitz plot (Supplementary Fig. 1a). While the Dalitz plot for lower KER region (6.0 - 6.8 eV) is located in the same region but exhibits a more broad distribution (Supplementary Fig. 1b). This suggests that the triangular breakup is still the dominant dissociation mechanism for the different KER regions. The underlying reason of the diffuse distribution in Supplementary Fig. 1b may be attributed to the structural rearrangement of the trimer before the ICD decay, in particular for the dSI+ICD channel. As in this process, the intermediate $C_6H_6^+ \cdot C_6H_6^{+*} \cdot C_6H_6$ state enable the increasing of intermolecular distance of two cations owing to the Coulomb repulsive force in the trimers, leading to a lower KER of the final three-body Coulomb explosion channel ($C_6H_6^+ + C_6H_6^+ + C_6H_6^+$). The KER difference can be used to estimate the lifetime of the ICD process, which is discussed in the following section. In the strong field fs laser experiment, the inner-valence ionization is a minor process, consequently, the ionization process similar to dSI+ICD is absent, resulting in the absence of low-energy KER (6.0 - 6.8 eV).

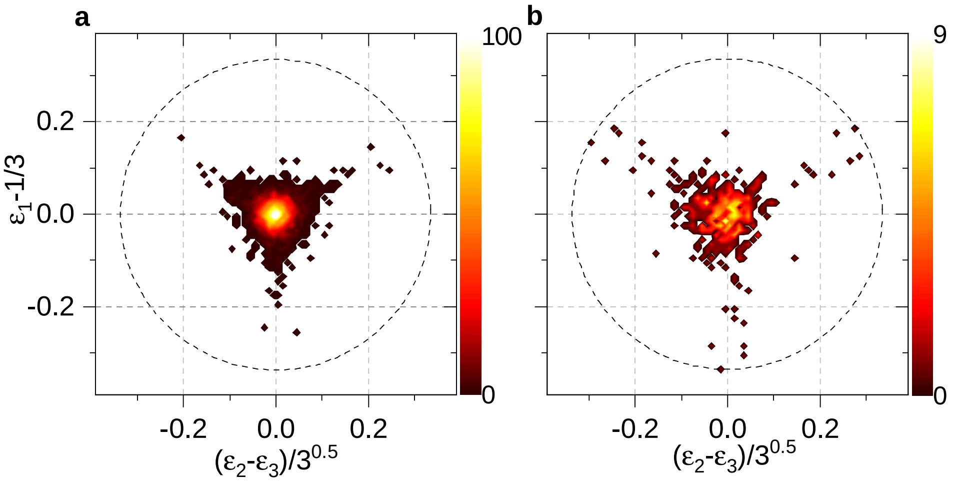

Supplementary Fig. 1. The experimental Dalitz plots of KER in the 6.8 - 9.0 eV (a) and 6.0 - 6.8 eV (b) regions. The color bar is linear with arbitrary units. Source data are provided as a Source Data file.

II. Supplementary Note 2

The Newton diagrams with a momentum range of ± 8 (arb. units) are presented in Supplementary Fig. 2 to visualize generally the three-body dissociation dynamics, in particular for the results where the slow ion is considered as a reference. The calculations with the cyclic and PD conformers agree well with the experimental patterns. In addition, the diagrams for PDT, PDS, ST, S, T1, and T2 conformers show more structures at larger momenta (> 3 arb. units) as the slow ions are considered as references. These structures are far from the range of the experimental patterns. Overall, the present results indicate that the cyclic trimer is the dominant structure of the benzene trimer with a small fraction of the PD conformer. While the contributions of the other conformers are minor in the present supersonic jet experiment.

III. Supplementary Note 3

In the dSI+ICD process, after the initial dSI reaction the intermolecular distance between two singly ionized benzene molecules is increasing under the action of the two-body Coulomb repulsion until the third benzene is ionized by ICD leading to three-body Coulomb explosion. Thus, the KER is determined as the sum of the kinetic energies of two $C_6H_6{}^+$ cations prior to ICD plus the remaining Coulomb energy of all three $C_6H_6{}^+$ cations, i.e.,

$$\mathrm{E}_{\mathrm{KER}}(t) = \mathrm{E}_{\mathrm{kin}}(t) + \mathrm{E}_{\mathrm{CE}}(t) \tag{1}$$

where the kinetic energies of two cations $\mathrm{E}_{\mathrm{kin}}$ and the Coulomb energy of all three cations following ICD (E_{CE}) can be calculated as:

$$\mathrm{E}_{\mathrm{kin}}(t) = \frac{q_1 q_2}{R_\mathrm{e}} - \frac{q_1 q_2}{R_1(t)} \tag{2}$$

$$\mathrm{E}_{\mathrm{CE}}(t) = \frac{q_1 q_2}{R_1(t)} + \frac{q_1 q_3}{R_2(t)} + \frac{q_2 q_3}{R_2(t)} \tag{3}$$

Here R_e is the equilibrium intermolecular distance, $R_1(t)$ is the distance between two $C_6H_6{}^+$ cations and $R_2(t)$ is the distance between the neutral C_6H_6 and $C_6H_6{}^+$ cation. The charge q_i with i = 1, 2, 3 is equal to +1. In the classical model, the $C_6H_6{}^+$ cation is treated as point charge neglecting its rotation and vibration. Since the dissociation mainly originates

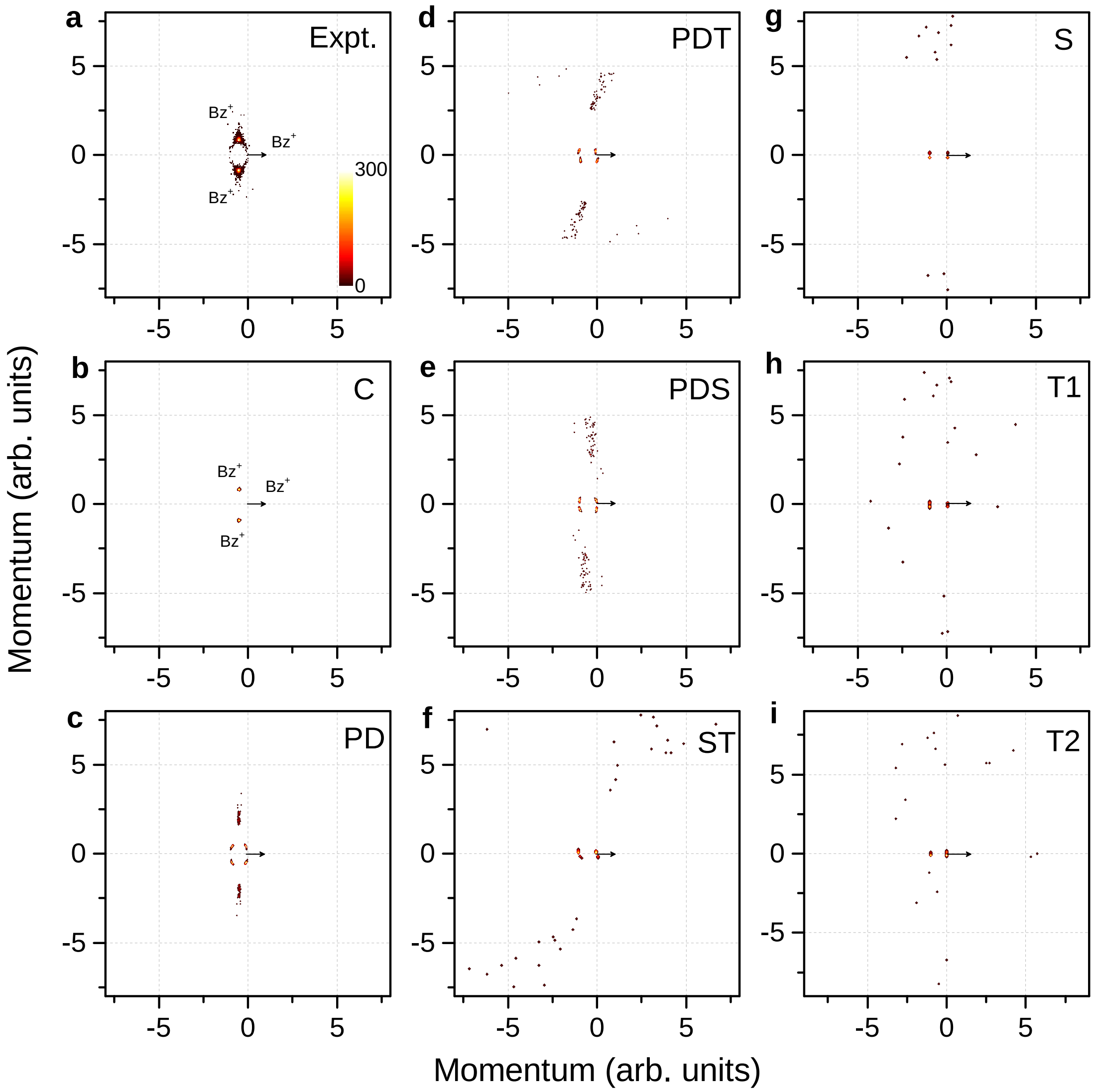

Supplementary Fig. 2. Experimental Newton diagrams and AIMD calculations for the three-body dissociation process. a, Experiment. b-i Calculations for eight different conformers of the benzene trimer: C-trimer (b), PD (c), PDT (d), PDS (e), ST (f), S (g), T1 (h) and T2 (i) conformers. The black arrows denote randomly the momentum of one of the three ${C_6H_6}^+$ since they are indistinguishable. The color bar is linear with arbitrary units. Source data are provided as a Source Data file.

from the regular triangle structure, $R_1(t)$ and $R_2(t)$ fulfill the following equation:

$$R_2(t)^2 - \frac{1}{4} \times R_1(t)^2 = (\frac{\sqrt{3}}{2} \times R_\mathrm{e})^2 \tag{4}$$

Assuming the distance between two ions is increasing by a sufficiently small spatial steps $\mathit{\Delta} R$ (we use a value of 0.001 Å in our numerical calculations), see the inset in Supple-

mentary Fig. 3, then we can determine the ion velocity $\upsilon(R)$ and the time interval $\Delta t(R)$ corresponding to ΔR as:

$$\upsilon(R) = \sqrt{2\mathrm{E_{kin}}/\mu} \tag{5}$$

$$\Delta t(R) = \frac{-\upsilon(R) + \sqrt{\upsilon^2(R) + 2a(R)\Delta R}}{a(R)} \tag{6}$$

Furthermore, we obtain the acceleration a_n

$$a_\mathrm{n} = -\frac{1}{\mu} \times \frac{V(R_\mathrm{n}) - V(R_{\mathrm{n}-1})}{\Delta R} \tag{7}$$

and

$$\Delta t_\mathrm{n}(R_\mathrm{n}) = \frac{-\upsilon(R_{\mathrm{n}-1}) + \sqrt{\upsilon^2(R_{\mathrm{n}-1}) + 2 \times a_\mathrm{n} \times \Delta R}}{a_\mathrm{n}} \tag{8}$$

where μ=$m_1 m_2/(m_1 + m_2)$ is the reduced mass of two $C_6H_6{}^+$ cations, $V(R)$ is the potential energy at the distance R. Thus, we can plot the curve ΔKER(t) = KER(t) − KER(t_0) in Supplementary Fig. 3. According to the KER difference between the main peak (7.4 eV) and the small shoulder (6.5 eV) shown in Fig. 2a of the Main Article, we can estimate a possible upper limit of the ICD lifetime in the dSI+ICD channel, which amounts to around 239 fs.

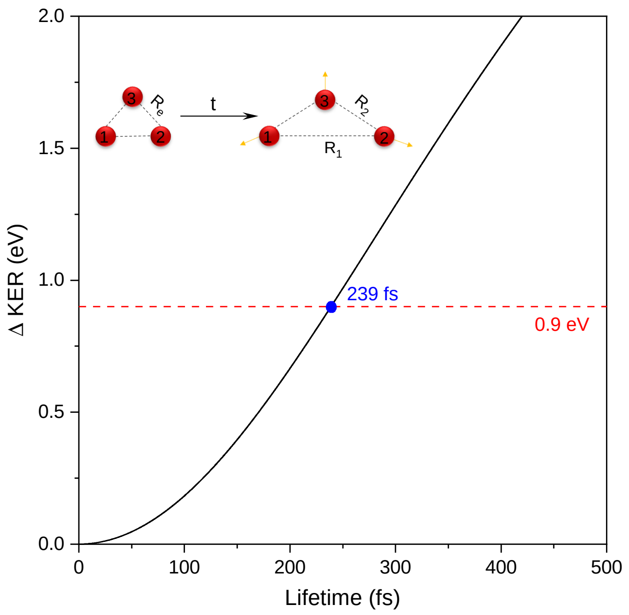

Supplementary Fig. 3. The correlation curve between the KER difference ΔKER and the ICD lifetime (0−500 fs). For the KER difference of 0−0.9 eV, the corresponding ICD lifetime can be estimated to be < 239 fs. Source data are provided as a Source Data file.

IV. Supplementary Note 4

The fragmentation dynamics of the three-body Coulomb explosion process of Thymine(T)-adenine(A)-thymine (T·A·T) trimer are shown in Supplementary Fig. 4. The calculated KERs are presented in Supplementary Figs. 4a and 4c for the PD (SS) and cyclic (TT) conformers of the trimers, respectively, where the former exhibits a single-peak structure centered at around 7.8 eV, while the latter shifts to a lower value at 6.5 eV. The real-time molecular motion for two conformers are presented in Supplementary Figs. 4b and 4d as molecular snapshots from $0 - 500$ fs, which are starting from the SS and TT geometries of the trimers. Both of them reveal concerted three-body dissociation process similar to that of the benzene trimer, and it is worth noting that the three cations exhibit significant rotation during the dissociation process, indicating that a part of the Coulomb potential is converted to the rotational energy of fragment ions during the Coulomb explosion. The further calculations of DNA bases trimers suggest that the concerted fragmentation mechanism can be a general phenomenon in biological systems. Due to the high presence of aromatic trimers with the cyclic and PD structures in proteins and DNA [1–3], the results obtained in this study could have important implications for radiation damage to biological matter.

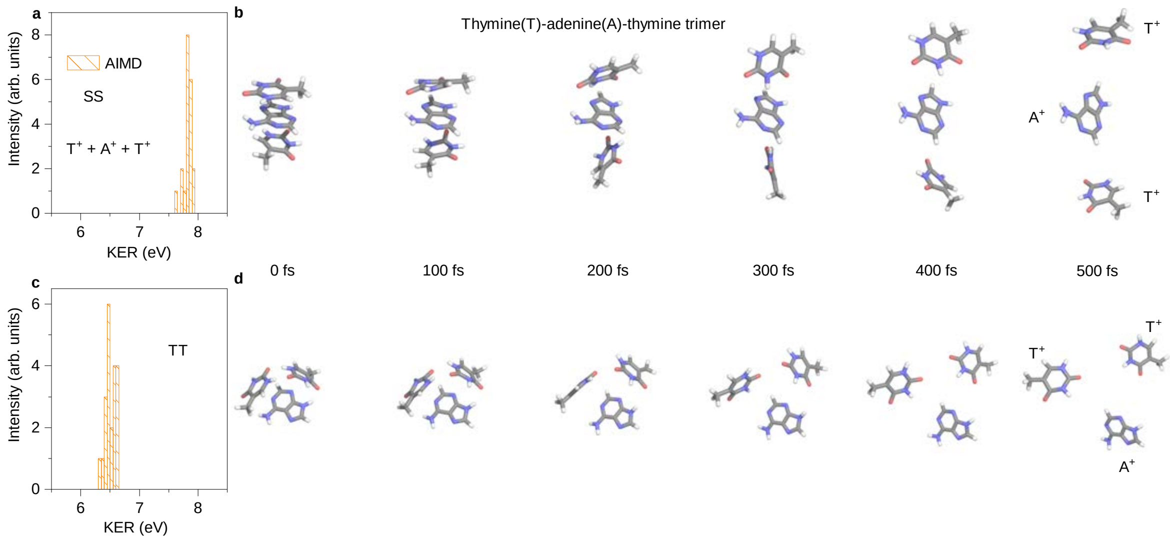

Supplementary Fig. 4. The calculated KER for T·A·T trimer of PD-type (SS) structure (a) and cyclic (TT) structure (c). The molecular snapshots of PD trimer calculations (b) and cyclic conformer calculations (d) from 0 − 500 fs. Source data in Supplementary Fig. 4a, c are provided as a source data file.

Supplementary References

[1] Burley, S. K. & Petsko, G. A. Aromatic-aromatic interaction: A mechanism of protein structure stabilization. *Science* **229**, 23–28 (1985). URL https://doi.org/10.1126/science.3892686.

[2] Sathiyashivan, S. D. *et al.* Steric group enforced aromatic cyclic trimer conformer in tripodal molecules. *RSC Advances* **5**, 74705–74711 (2015). URL https://doi.org/10.1039/C5RA05151G.

[3] Lanzarotti, E., Biekofsky, R. R., Estrin, D. A., Marti, M. A. & Turjanski, A. G. Aromatic–aromatic interactions in proteins: Beyond the dimer. *Journal of Chemical Information and Modeling* **51**, 1623–1633 (2011). URL https://doi.org/10.1021/ci200062e.
